# Role of Cellular Senescence in IUGR: Impact on Fetal Morbidity and Development

**DOI:** 10.3390/cells14141097

**Published:** 2025-07-17

**Authors:** Aliabbas Zia, Faezeh Sahebdel, Yosra Er-Reguyeg, Michel Desjarlais, Jean-Clement Mars, Gregory A. Lodygensky, Sylvain Chemtob

**Affiliations:** 1 Research Center of Centre Hospitalier Universitaire Sainte-Justine, Montreal, QC H3T 1C5, Canada; aliabbas.zia@umontreal.ca (A.Z.); micheldesjarlais@gmail.com (M.D.); mars.jeanclement@gmail.com (J.-C.M.); ga.lodygensky@umontreal.ca (G.A.L.); 2 Department of Pharmacology, Université de Montréal, Montreal, QC H3T 1J4, Canada; 3 Department of Physical Medicine and Rehabilitation, Miller School of Medicine, University of Miami, Miami, FL 33136, USA; faezeh.sahebdel@gmail.com; 4 Faculty of Medicine, Laval University, Québec City, QC G1V 0A6, Canada; yosra.er-reguyeg.1@ulaval.ca; 5 Ophthalmology Department, Hôpital Maisonneuve-Rosemont Research Center, Montreal, QC H1T 2M4, Canada; 6 Department of Pediatrics, Université de Montréal, Montréal, QC H3T 1C5, Canada

**Keywords:** IUGR, senescence, oxidative stress, Rytvela, development

## Abstract

Intrauterine growth restriction (IUGR) is a critical challenge in perinatal medicine and is associated with significant morbidity and mortality. This review explores the intricate involvement of early developmental senescence in IUGR. We highlight the dual role of cellular senescence in both normal development and pathological conditions, emphasizing the need for further research to elucidate these mechanisms and develop targeted interventions. We discuss how oxidative stress and mitochondrial dysfunction affect senescence determinants. We present emerging therapeutic strategies aimed at targeting senescence and inflammation in the placenta. We also introduce Rytvela, an interleukin-1 (IL-1) receptor modulator developed in our laboratory, which selectively attenuates pro-inflammatory signaling while preserving essential immune responses, which in turn mitigate senescence. By addressing senescence-related dysfunctions, such interventions may improve placental performance and fetal outcomes, opening up new directions for the clinical management of IUGR.

## 1. Introduction

Intrauterine growth restriction (IUGR), alternatively termed fetal growth restriction, defined as the condition in which the growth of the fetus falls below the tenth percentile. The etiology of IUGR encompasses genetic and environmental conditions linked to placental, fetal, and maternal anomalies. This condition serves as a primary cause for a majority of elective late-preterm deliveries [1,2,3]. This review focuses primarily on IUGR associated with placental compromise, while recognizing that genetic and infectious factors may also contribute to its multifactorial pathophysiology. IUGR is linked to a high number of childhood and adult morbidities and mortalities, a concept initially articulated by Barker et al. [4] as the Developmental Origins of Adult Health and Disease, positing that adverse conditions during critical developmental periods play a pivotal role in biological programming, ultimately affecting the rate of aging [5,6] and increasing the risk of vascular disorders.

Aside from genetic syndromes associated with IUGR, and maternal conditions such as hypertension, kidney disease, intrauterine infections, as well as smoking and alcohol consumption, which can curtail growth factor actions, the mechanisms underlying placental insufficiency and the associated fetal growth compromise are not well understood, and has resulted in a dearth of therapeutic interventions. Of relevance here is that cellular senescence—irreversible cell cycle arrest—was initially described in the context of replicative senescence by Hayflick in 1965 [7]. In this review, we focus on stress-induced premature senescence, which can be triggered by various stressors and is clearly observed under these conditions [1,8]. The interplay between senescence and disrupted physiological systems involving the endoplasmic reticulum (ER) and oxidative stress in IUGR is elaborated in this review.

The rate of children born with a low birth weight is approximately six times higher in developing countries than in developed countries [9]. Low birth weight (defined as <2500 g) affects ~16% of all newborns born in developing countries (or ~20 million infants) each year [9,10]; thus, ~30 million infants suffer from IUGR every year [9]. The burden of IUGR is concentrated mainly in Asia, which accounts for nearly 75% of all affected infants in developing countries [11].

IUGR is associated with significant morbidity, resulting in short-term meconium aspiration, hypoglycemia, polycythemia, intrapartum asphyxia, and stillbirth [12,13]. In the longer term, IUGR as a risk factor for preterm birth affects body growth and neurodevelopment, and is associated with behavioral and social disorders. In this regard, IUGR can lead to impaired brain reserve, due to structural and size deficits, which may contribute to an increased risk of cognitive impairment and dementia in aging populations, as suggested by epidemiological data [14,15]. IUGR is also associated with a greater predisposition to adult-onset obesity, hypertension, hypercholesterolemia, cardiovascular disease, and type 2 diabetes [16,17]. Hence, IUGR can lead to acute, sub-acute, and long-term complications for the offspring.

The biological trade-off involved in overcoming developmental growth restriction may induce cellular stress, such as stress due to an increased replication burden or metabolic imbalance, thereby predisposing cells to the early onset of senescence. Senescence can be prematurely triggered in response to various cellular stressors and damaging processes—including inflammation, oxidative stress, DNA damage, and telomere shortening—which can activate downstream signaling pathways that converge on senescence programs. These factors are likely contributors to IUGR. Central to this process is the induction of key proteins that affect the cell cycle and cell fate, such as those belonging to the p16/RB and p53/p21 tumor-suppressor axes; these proteins play a crucial role in curtailing proliferation and contribute to the senescent state [18,19]. Senescent cells are commonly associated with pathological conditions, particularly in aged tissues and within the tumor microenvironment, where they may arise in response to intrinsic stress or therapeutic interventions. Beyond its involvement in pathological conditions in adults, senescence has a crucial physiological role during embryogenesis, actively participating in tissue growth and organ patterning [20,21]. Throughout embryonic development, cells displaying senescence-related features are strategically located in specific areas and at crucial developmental stages. These include the mesonephros, the hindbrain roof plate (neural roof plate), the apical ectodermal ridge (AER) of the limb, and the endolymphatic sac [22,23]. Controlled induction of senescence plays a major role in determining cell fate and influences tissue development, with the subsequent effective elimination of these cells aiding in the process of tissue remodeling [23,24]. Notably, developmental senescent cells have an expression profile similar to that seen in oncogene-induced senescence, particularly in relation to the senescence-associated secretory phenotype (SASP). This intriguing parallel suggests that embryonic senescent cells leverage SASP components to modulate spatiotemporal patterning during development [22].

Abundant evidence also points to a role of senescence in pathological conditions including those related to early life development [21,25]. Recent investigations have proposed that the shift from a non-labor to labor status at term is associated with the senescence of cells in the placental membrane, accompanied by SASP factor production [26,27]. Studies indicate that decidual senescence and the associated inflammation through the SASP play a significant role in spontaneous labor. Physiologically, cellular senescence in the fetus occurs during normal pregnancies, particularly at term, and contributes to the onset of spontaneous labor. However, when decidual senescence becomes aberrant, it can result in fetal loss and preterm birth [28,29]. The molecular and cellular mechanisms underlying detrimental pregnancy complications have not been fully elucidated. As mistimed induction of senescence is implicated in various adult diseases, there is a growing interest in exploring whether aberrant senescence might also be involved in developmental disorders, including IUGR. This review aims to provide a comprehensive overview of the role of stress-induced premature senescence in the pathophysiology of IUGR, with particular attention to its interaction with oxidative stress, mitochondrial dysfunction, and ER stress. It also highlights how these interconnected mechanisms may influence fetal development and contribute to long-term health consequences.

## 2. Telomere Shortening and IUGR

One of the critical molecular pathways implicated in IUGR is telomere dynamics which partakes in senescence. Telomeres, specialized nucleoprotein structures situated at the ends of chromosomes, serve as guardians, shielding chromosomes from degradation and end-to-end fusion. Telomeres, which maintain chromosomal integrity, are particularly vulnerable to intrauterine stressors such as oxidative damage, inflammation, and placental insufficiency [30]. These protective structures undergo progressive shortening with each round of DNA replication and in response to environmental stressors [31]. Critically short telomeres are associated with the activation of a DNA damage response (DDR), which can lead to cell cycle arrest, apoptosis, and genomic instability. Emerging evidence suggests that telomere shortening in fetal and placental cells may serve as a key mediator of growth restriction, influencing both immediate developmental outcomes and long-term disease risk [30,32]. The following section explores the role of telomere attrition in IUGR and its broader implications for fetal morbidity and postnatal health. Critically short and uncapped telomeres tend to undergo end-to-end fusion, which promotes chromosomal aggregates and contributes to genomic instability [31,33]. Crucially, the maintenance of telomere length is orchestrated by the enzyme telomerase, a ribonucleic protein complex comprising a catalytic domain (human telomerase reverse transcriptase: hTERT) and an RNA template (human telomerase RNA: hTR), which includes an 11-nucleotide template region for telomere synthesis as well as additional domains essential for structural integrity and enzymatic function. Telomerase extends chromosomes via the addition of TTAGGG sequences to their ends [34]. Telomere maintenance mechanisms encompass both elongation by telomerase and an alternative process known as recombination-mediated lengthening, which involves the extension or capture of telomeres. Among these, telomere capture refers to a recombination-based mechanism in which a telomeric sequence from another chromosome is acquired to stabilize chromosome ends—which is observed in cells lacking telomerase or following telomeric dysfunction. This phenomenon is related to the alternative lengthening of telomeres (ALT) pathway, which relies on homologous recombination to elongate telomeres and is notably active during early development or in certain cancer cells [35].

Telomeres play a pivotal role in influencing health, aging dynamics, longevity, and the development of various genetic disorders. Also, telomere biology has been proposed as a mechanism linking fetal intrauterine conditions to later health outcomes [36,37]. Recent findings indicate that fetal telomere length is linked to prenatal factors such as maternal exposure to tobacco smoke, stress, medical conditions, and disturbances in nutrition and sleep during pregnancy [38,39]. Recent studies have shown that impaired telomere homeostasis has been implicated in the pathophysiology of IUGR, potentially influencing fetal development and contributing to the onset of diseases in adulthood. Babies with IUGR typically have a higher risk of developing metabolic, cardiovascular, and neurological disorders in adulthood, conditions often associated with short telomeres [30,32]. An intriguing study in young men by Laganovic et al. [40] showed faster telomere shortening in all premature birth groups in comparison to control groups. Additionally, placentas from pregnancies in women affected by IUGR exhibited shorter telomere length [41], supporting the role of telomere shortening and telomerase activity resulting from placental insufficiency in IUGR. The long-term consequences of placental damage can thus be carried into adulthood, contributing to conditions such as type 2 diabetes mellitus, metabolic syndrome, coronary artery disease, and stroke [42].

In this regard, an assessment of hTERT mRNA revealed a reduction in IUGR placental size, implying that the diminished hTERT protein levels observed in these placentas stem from decreased mRNA levels, resulting in reduced protein production [32]. A notable increase in telomere capture in IUGR trophoblasts in comparison to control tissues was also observed. These findings propose a potential compensatory mechanism in IUGR placentas to counteract the effects of shortened telomeres and maintain telomere homeostasis [32].

A decrease in the level of telomerase RNA component (TERC—non-coding RNA) in IUGR placentas has also been reported [43], resulting in a reduction in the transcription of the TERC gene on chromosome 3, along with similar copy number losses affecting other chromosomal regions. This observation suggests that placental pathology in IUGR reflects broad genomic instability rather than localized damage. The authors proposed the possibility that TERC amplification, which typically occurs in normal trophoblasts as part of placental development through pregnancy, may be reduced or absent in pregnancies affected by IUGR, consistent with hypoxic damage to the trophoblast [43]. Chronic hypoxia causes oxidative stress and HIF-1α-mediated repression that disrupt normal TERC amplification in trophoblasts. This occurs through transcriptional suppression, oxidative DNA damage at the TERC locus, and impaired epigenetic regulation, collectively contributing to telomere dysfunction and genomic instability [44].

In models of a pan-gestational low-protein diet followed by normal postnatal nutrition, the offspring exhibited an increased obesity risk [45], which was associated with shorter telomeres [46,47]. This simulates protein restriction-induced fetal growth restriction followed by postnatal catch-up growth—a sequence that was found to promote telomere shortening [47]. On the other hand, mice that do not recover weight postnatally do not display telomere shortening [48], while those that recover present glucose intolerance by nine months of age. Importantly, only the subjects exhibiting fast catch-up growth displayed telomere shortening. This likely reflects the increased replicative demands during accelerated postnatal growth; this oxidative stress during catch-up both directly damages telomeres and redistributes TERT to mitochondria (reducing nuclear telomerase activity) [49], and the combined effects of these processes overwhelming the telomere maintenance mechanisms.

Studies have suggested that in mice born with a low birth weight due to IUGR that subsequently experience rapid postnatal weight gain, the telomere reduction reflects the increase in cell divisions and oxidative stress accompanying the rapid catch-up growth, rather than representing an adaptive response. While inflammatory and endocrine pathways may contribute secondarily by altering oxidative stress levels, the dominant drivers remain the replicative demands and accelerated postnatal growth [46,48].

The notion of stress impacting telomere length in humans has been proposed [50,51,52]. The findings from these studies reinforce the connection between telomere length and lifespan and underscore the importance of identifying factors that influence telomere length in early life [53].

### 2.1. Telomere Shortening in IUGR-Associated Preeclampsia (PE)

**Figure 1:**
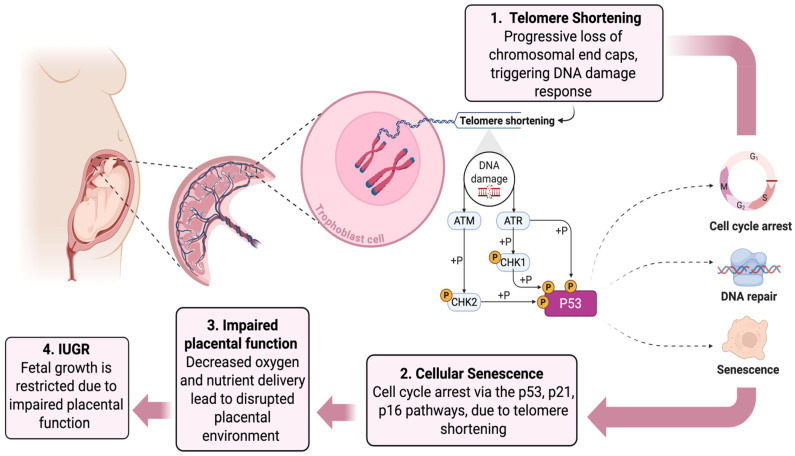
Telomere shortening contributes to placental dysfunction and IUGR. Progressive telomere shortening in trophoblast cells activates a DNA damage response (DDR), mediated by the activation of Ataxia Telangiectasia Mutated (ATM) and ATM and Rad3-Related (ATR) kinases. This leads to the activation of p53, inducing cell cycle arrest and cellular senescence (Steps 1–2). The accumulation of senescent cells results in impaired placental function, reducing oxygen and nutrient delivery to the fetus (Step 3), and ultimately causing the restricted fetal growth seen in IUGR (Step 4). This sequence provides a mechanistic link between cellular senescence processes and adverse pregnancy outcomes, specifically IUGR. Created with www.BioRender.com.

PE is characterized by maternal proteinuria and hypertension, wherein altered placental implantation is linked to placental insufficiency and is thus a major cause of IUGR [54]. This complication significantly impacts the short-term and long-term health outcomes for the mother and fetus and can occur concurrently or independently. It has been found that telomere length was notably shorter in trophoblasts of placentas from pregnancies with PE with or without IUGR than in trophoblasts of normal pregnancies [55]. Additionally, hTERT, the catalytic domain of the telomerase enzyme, was under-expressed in the study groups compared to the controls, aligning with the observation of shortened telomeres in these groups. Notably, significant telomere aggregates were observed in the placentas affected by PE, as well as in those affected by both PE and IUGR, but not in placentas from pregnancies complicated solely by IUGR. These results suggest distinct telomere dynamics in the context of PE, possibly indicating unique molecular mechanisms underlying telomere maintenance in this condition compared to IUGR alone [55], as illustrated in Figure 1.

In human trophoblasts affected by PE, increased hTR copy numbers were observed along with increased expression of the telomere capture mechanism components [56]. Telomere capture is considered to be a mechanism to compensate for the shortening of telomere and senescence. Notably, no telomere shortening or an increase in the formation of aggregates was detected in cord blood cells from pregnancies with PE. There was also no increase in senescence-associated heterochromatin foci; the hTR copy numbers remained unaltered, indicating that telomere capture was not enhanced in cord blood cells. Contrary to the altered telomere homeostasis in PE trophoblasts, no differences were detected in cord blood cells from pregnancies affected by PE in comparison to control samples [35,56]. The absence of telomere length changes in the peripheral blood of women with PE was also observed by other investigators [57]. These findings suggest that the impact of telomeres on PE pathogenesis is specific to the placenta, and telomere homeostasis remains unchanged in the cells and tissues of the fetus and mother. While the placenta normally provides protection against environmental stressors, this capacity may be compromised in IUGR, permitting both endogenous and exogenous stressors to collectively accelerate telomere shortening.

### 2.2. Insulin-like Growth Factor (IGF) and Telomere Length

The insulin/insulin-like growth factor (IGF) signaling pathway is a fundamental and evolutionarily conserved modulator of growth and aging [58]. IGF-1 signaling plays a crucial role in influencing mammalian aging and related diseases [58,59]. Throughout pregnancy, IGF-1 signaling serves as a key regulator for the development of the fetus and placenta [60,61]. Notably, higher tissue levels of IGF-1 are associated with longer telomeres [58]; conversely, telomere shortening and lower levels of IGF-1 are hallmarks of aging [62]. Maternal levels of IGF-1 exhibit a dynamic pattern: declining in the first trimester, experiencing a notable increase of over 40% between weeks 17 and 24 [63], and subsequently decreasing rapidly to the pre-labor baseline after delivery [64]. This fluctuation suggests a finely tuned regulation at the level of IGF-1 in response to the body’s changing needs during pregnancy. In both humans and rodents, transcripts of IGF-2 are present in all fetal tissues, with their transcripts declining early in the postnatal age [65].

## 3. Oxidative Stress, Senescence, and IUGR

Alongside telomere shortening—which may arise as a consequence of oxidative stress—cellular stressors also play a crucial role in the pathophysiology of IUGR. In this context, oxidative stress, a significant source of DNA damage and cellular dysfunction, has been increasingly defined as a key factor influencing both telomere dynamics and fetal development [66]. The excessive production of reactive oxygen species (ROS) not only accelerates telomere attrition but also induces widespread molecular damage, triggering cellular senescence and impairing placental function [66,67]. The following section reviews the intricate relationship between oxidative stress, senescence, and IUGR, highlighting the impact of mitochondrial dysfunction and ER stress on fetal growth restriction.

Oxidative stress involves an increase in intracellular ROS, which disrupts lipids, proteins, and DNA [68]. Studies have reported a link between oxidative and ER stress in numerous pathologic conditions [69]. Mitochondria, employing oxygen as the ultimate acceptor of electrons in aerobic respiration, generate ROS and ROS by-products [70]. While limited ROS levels are essential for certain enzymatic reactions and signaling pathways during a balanced redox state, excess ROS levels lead to oxidative damage to macromolecules [71,72]. Oxidative stress also triggers ER stress, leading to the accumulation of misfolded proteins within the ER, which is detrimental to cell survival as ER stress hampers the production of functional proteins which can ultimately result in apoptosis [73,74].

### 3.1. Mitochondrial Dysfunction and IUGR

Disturbances in mitochondrial homeostasis stand out as a pivotal hallmark of cellular senescence [75]. Aging is commonly associated with a gradual decline in mitochondrial integrity [72], leading to the increased generation and leakage of ROS. This is associated with the depolarization of the inner membrane, resulting in impaired mitochondrial electron transport and altered metabolic processes. Such alterations in mitochondrial function can trigger the activation of pro-senescence p53-p21 and/or p16-pRB signaling cascades [72,76]. In IUGR-induced metabolic syndrome, the liver appears to be particularly vulnerable, with evidence pointing to hepatic oxidative stress and mitochondrial challenges in offspring with growth restriction. For instance, in a pig IUGR model, neonates with growth restriction exhibited higher amounts of hepatic alpha-1-acid glycoprotein (AGP) at birth, indicative of oxidative stress [77]. These newborns also showed higher amounts of complex IV protein in the electron transport chain, indicating a potential reduction in ATP availability due to increased ATP hydrolysis [77]. In a rat model subjected to caloric restriction (maternal caloric intake was reduced by 50% from gestational day 11 to postnatal day 21), decreased glutathione levels and increased levels of the lipid peroxidation marker 4-hydroxynonenol (4HNE) were observed at three weeks of age [78]. Notably, in offspring that transitioned from a low-protein diet to a balanced diet at weaning, sustained high levels of 4-HNE were observed into adulthood, despite steady antioxidant levels. This suggests that it is not intrauterine restriction alone, but rather the postnatal shift to a normal diet following early caloric restriction that contributes to long-term oxidative stress [78].

The pancreas is also affected by IUGR-induced metabolic syndrome, manifesting impaired insulin secretion due to β-cell dysfunction at birth, which persists into childhood and adulthood [79,80,81]. Oxidative stress has been implicated in this outcome, with IUGR pancreatic islets experiencing an increase in ROS levels [82]. In pre-clinical IUGR models, isolated islets from the first week of life displayed elevated ROS levels, which further increased at 15 weeks [83]. Concurrently, mitochondrial citrate synthase activity and the function of complexes I and III were diminished at week 15, resulting in reduced ATP production throughout fetal and postnatal life [81,84]. Alterations in the protein level and activity of SOD, catalase, and glutathione peroxidase in the protein-restricted pancreas reinforce the imbalances in redox potential [84].

### 3.2. Oxidative Stress and Senescence in the Placenta

Cellular senescence is elicited in response to various intrinsic and extrinsic stressors. Pregnancy serves as a conduit for stress signals, including oxidative and hypoxic stress, which can influence the development of the placenta. Pregnancy and the early postnatal period represent metabolically demanding phases for both the mother and offspring [85]. IUGR increases mitochondrial dysfunction, which in turn compromises energy production, and, through a vicious cycle, curtails fetal growth [82]. Studies indicate that mothers of infants with growth restriction exhibit higher levels of ROS and a reduction in antioxidant levels in their blood, and tissues from IUGR offspring display oxidative stress [86]. This involves not only elevated ROS levels but also changes in antioxidant enzyme levels, increased lipid peroxidation, and reduced ATP synthesis [82,86]. It is thus crucial to recognize that hypoxic and oxidative stresses, at certain stages of development, have the potential to hinder proper placental integrity, consequently contributing to IUGR and PE [66,67,87].

Decidual senescence is associated with spontaneous parturition [28]. The increased oxidative stress that stems from IUGR and/or uterine stretching may induce senescence and consequently, labor. For instance, increased levels of 8-hydroxy-2′-deoxyguanosine (8-OHdG), which is associated with oxidative damage to DNA, can induce senescence. Importantly, high levels of 8-OHdG in the placenta have been linked to various adverse pregnancy outcomes, including PE, IUGR, and fetal death [88]. Additionally, High Mobility Group Box 1 (HMGB1), a damage-associated molecular marker for inflammation intertwined with oxidative stress, has been linked to preterm birth and pPROM [89]. HMGB1 increases the secretion of inflammatory cytokines and activates enzymes involved in collagen remodeling, potentially contributing to labor induction [90], and activates the cell fate guidance molecules p53, p21, and p38 MAPK, which are present at term, the preterm stage, and preterm pregnancy rupture of the membrane (pPROM) associated with IUGR, implying a prominent role for cellular senescence in compromising normal gestation [91].

Investigations comparing non-laboring to laboring placental membranes in normal-term pregnancies revealed biochemical and histological changes associated with senescence. Menon [26] showed a comparable amount of oxidative damage markers (8-OHdG), HMGB1, and senescence-associated telomere shortening in the term and preterm subgroups, as well as among the preterm subgroups with ruptured membranes. Significant correlations were found between HMGB1 and 8-OHdG levels; despite similar telomere lengths between preterm and term placentas, earlier telomere shortening was observed in those associated with preterm labor, suggesting that the critical telomere length necessary for labor initiation may have been achieved in both the preterm and term placentas [26]. Overall, these findings underscore the significance of cellular senescence and associated oxidative/inflammatory processes in interfering with gestational uterine quiescence.

The rationale presented above on oxidative stress, inflammation, and senescence in IUGR can also be extended to IUGR-predisposed stillbirth [92]. It is hypothesized that during late pregnancy, as the fetal requirement for nutrients and oxygen increases, the placenta may become stressed by its limited capacity to transfer oxygen and nutrients to the fetus. This stress can trigger the generation of ROS, culminating in oxidative damage and subsequent aging of placental tissue [93,94], which can compromise fetal growth, resulting in IUGR. The risk of fetal demise escalates significantly in late pregnancy, particularly after 41 weeks of gestation, implying that placental aging has a pivotal role in the clinical manifestations of this complication [95,96]. In this regard, Ferrari et al. [97] revealed telomere shortening in the placenta associated with unexplained fetal death. Moreover, the length of telomeres in placentas from fetuses that died closely resembled that observed with pPROM, which were, as expected, shorter than the telomeres of placentas from other preterm births [97], suggesting that senescence markers are both indicators and effectors of placental/fetal demise.

**Figure 2:**
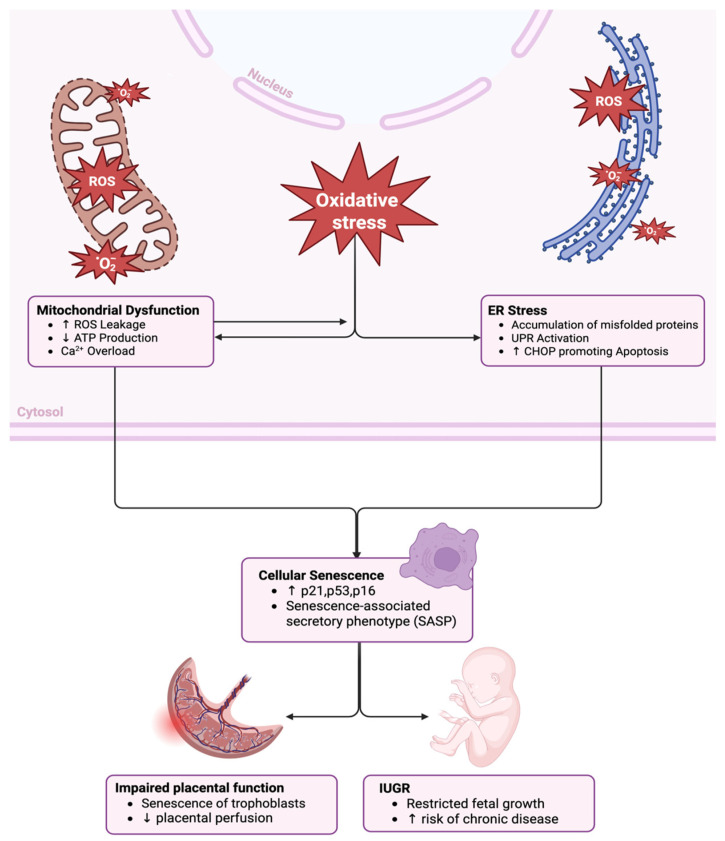
Oxidative stress-driven mitochondrial and endoplasmic reticulum (ER) dysfunction contributes to IUGR. Oxidative stress leads to mitochondrial dysfunction—characterized by increased reactive oxygen species (ROS) leakage, decreased ATP production, and calcium overload—and to ER stress, marked by protein misfolding, unfolded protein response (UPR) activation, and CHOP-mediated apoptosis. These stress responses trigger cellular senescence through the upregulation of p53, p21, and p16, along with the secretion of pro-inflammatory factors (SASP). The senescence of placental trophoblasts compromises placental perfusion and function, ultimately resulting in restricted fetal growth and an increased risk of chronic diseases in the offspring. Created with www.BioRender.com.

In addition to affecting body growth as elaborated above, senescence has been detected in vulnerable newborn tissues exposed to oxidative stress such as the lungs, gut, and retina [98,99,100]. These observations were associated with a compromised ability of the tissues to repair themselves and grow, resulting in defective functions. Figure 2 schematically illustrates the sequence of oxidative stress/inflammation triggering senescence perturbing placental well-being and compromising fetal growth, resulting in IUGR.

### 3.3. Oxidative Stress-Triggered ER Stress, Unfolded Proteins, and IUGR

Oxidative stress often coincides with ER stress. When the ER is unable to facilitate accurate protein folding, it results in the accumulation of unfolded or misfolded proteins in the ER lumen, leading to ER stress. The physical connection between mitochondria and the ER occurs at specialized locations known as the mitochondrial-associated ER membrane. These regions play an indirect role in ATP production and respond to ER signaling, particularly when the demands for protein folding are elevated [101]. ER stress is intricately linked to senescence pathways in various diseases. However, it remains unclear whether senescence actively triggers ER stress or if it arises as a consequence of ER stress [102]. Moreover, the extent of ER stress severity and its impact on the development of senescence outcomes remain ambiguous. Nonetheless, ER stress can occur concurrently with senescence and may contribute to the initiation or perpetuation of senescence characteristics [103].

ER stress is vital to cell function as an integrated stress response [104,105] during pregnancy and embryonic development [106]. Prolonged or excessive ER stress can be initiated by various cellular challenges, including oxidative stress, defective growth factors, disruptions in calcium homeostasis, a diminished amino acid supply, viral infections, compromised disulfide bond formation, and reduced N-linked glycosylation [100,107,108]. Given the association between ER stress and glucose intolerance, as observed postnatally in former IUGR offspring, it appears logical that tissues of IUGR subjects display an Unfolded Protein Response (UPR) associated with ER stress in organs crucial to glucose homeostasis such as the liver and pancreas, as ER stress can impair insulin signaling and glucose metabolism through disruption of cellular protein folding and inflammatory pathways [109]. Thus, catch-up growth in the postnatal period has been shown to induce ER stress, especially in the IUGR fetal liver [110]. In rodent models, it has been found that UPR activation occurring before or simultaneous to increased hepatic gluconeogenesis leads to glucose intolerance and compromised hepatic glycogen storage in later stages [110,111,112,113]. In this context, uteroplacental insufficiency has been found to result in elevated levels of ER stress markers in newborns and postnatally, along with increased amounts of gluconeogenic enzymes such as glucose-6-phosphatase catalytic subunit (G6P) and Phosphoenolpyruvate Carboxykinase 1 (PCK1) [113].

In unstressed cells, the activation of transmembrane proteins PERK, IRE1, and Atf6, which trigger ER stress, is hindered by the chaperone protein GRP78 (also referred to as Binding Immunoglobulin Protein [BiP]) via binding to their N-terminal domains in the ER lumen [114,115]. However, under conditions of ER stress, these transmembrane proteins are released from GRP78, initiating their oligomerization and activating targets that enhance an adaptive response [114]. This response involves an increased transcription of genes to enhance the ER’s folding capacity or facilitate protein degradation. In instances where the ER fails to effectively clear unfolded and misfolded proteins, the activation of C/EBP-homologous protein (CHOP) leads to cell death induced by ER stress [107,108]. A comparable UPR activation is also observed in the fetal pancreas when subjected to a low-protein diet [109]. Importantly, excess ER stress is an inducer of cell death in key metabolic organs, the liver, and the pancreas [116].

IUGR is often associated with a defective spiral artery supply of oxygen and nutrients to the placenta, which in turn causes ischemia–reperfusion injury. Such ischemic–reperfusion injury diminishes intracellular ATP production, which, as addressed above, leads to oxidative stress and ER stress in IUGR, particularly in cases of PE [109,110]. These changes affect the activation of the critical growth-associated signaling molecule AKT, which regulates cell proliferation and modulates protein synthesis through the mTOR and GSK-3 pathways. Accordingly, dysregulation of AKT-mTOR signaling, as observed in IUGR placentas, reduces the amount of mTOR, TSC2, and raptor proteins, which suppresses protein translation initiation and alters the elongation stage of protein synthesis, as evidenced by the increased phosphorylation of Eukaryotic Elongation Factor 2 Kinase (eEF2K) [117], which is robustly implicated in ER stress. Altogether, ER stress is intricately involved in processes associated with senescence and contributes to the aggravation of IUGR.

## 4. Detection of Senescence

### 4.1. Biomarkers as Novel Translational Directions

The development of non-invasive biomarkers of placental senescence is an exciting frontier in translational obstetrics. Circulating plasma exosomal miRNAs are associated with pregnancy complications of placental origin like IUGR and preterm birth [118,119]. A study by Hromadnikova et al. [120] assessed first-trimester circulating C19MC microRNAs for their ability to predict preeclampsia and IUGR. Among the six miRNAs tested (miR-517-5p, miR-516-5p, miR-518b, miR-520a-5p, miR-520h, and miR-525-5p), miR-517-5p showed the highest predictive performance for preeclampsia with the largest area under the curve (AUC) of 0.70, a sensitivity of 42.9%, and a specificity of 86.2%. When combined into a panel, the six microRNAs had improved specificity (90.8%) but limited sensitivity (20.6%), suggesting their utility as part of a broader biomarker strategy rather than standalone predictors. However, the same panel could not distinguish between pregnancies that later developed IUGR and those with normal outcomes, with no statistically significant differences in miRNA levels between the groups (*p* > 0.2 for all). Despite these limitations, the study highlights the potential of early circulating miRNA profiling as a promising avenue, warranting further investigation with refined biomarker panels and larger, IUGR-specific cohorts.

Methylation patterns of circulating cell-free DNA (*cfDNA*)—another indicator of pregnancy complications—show that DNA methylation in maternal blood is a promising target for IUGR screening [121,122,123]. Elevated levels of circulating *cf*DNA have also been observed in pregnancies complicated by IUGR, particularly in cases associated with placental insufficiency. Notably, this increase was present in patients both with and without preeclampsia, indicating that fetal DNA release is closely linked to placental dysfunction irrespective of the presence of hypertensive disease [124].

Senescence-related proteins (e.g., p16 ^INK4a^ and HMGB1) could serve as early indicators of placental aging and fetal compromise [26]. Early detection could enable timely therapeutic interventions and personalized care strategies.

### 4.2. Artificial Intelligence (AI) and Predictive Models in Precision Obstetric Medicine

Finally, the integration of artificial intelligence (AI)-driven models to predict IUGR and preterm birth risk based on imaging and multi-omics data—such as proteomic, transcriptomic, and metabolomic data—represents a transformative advance in precision obstetric care [125,126]. Recent computational frameworks, including machine learning (ML) and deep learning algorithms, have shown promising results in identifying complex, non-linear patterns associated with adverse pregnancy outcomes [127]. For instance, AI approaches trained on longitudinal clinical data and molecular signatures have successfully predicted preterm birth with increasing accuracy, enabling early risk stratification and clinical intervention [127,128,129]. ML models are being increasingly adopted as clinical decision support tools in obstetrics. Their predictive capabilities offer several advantages over traditional statistical approaches, particularly in handling complex and heterogeneous data types such as electrohysterogram (EHG) signals, electronic health records (EHRs), and transvaginal ultrasound imaging. By integrating these diverse inputs, ML models have demonstrated improved accuracy and robustness in predicting adverse pregnancy outcomes [130,131]. A recent study used data from over 8800 nulliparous women from the nuMoM2b cohort study to predict preterm birth based on routinely collected prenatal variables. Model performance improved across successive prenatal visits, with the AUC increasing from 0.62 at the first visit to 0.71 by the third, particularly when incorporating ultrasound parameters such as cervical length and the uterine artery pulsatility index. At the third visit, the model achieved sensitivities of 82.5% and 92.9% for very preterm and extreme preterm birth, respectively, underscoring the value of combining machine learning-based risk stratification with late-pregnancy ultrasound for improved clinical decision-making [132]. By incorporating emerging diagnostic/predictive biomarkers of cellular senescence into these models, clinicians could identify at-risk pregnancies before clinical symptoms arise, allowing for tailored therapeutic strategies to dramatically improve maternal–fetal outcomes using personalized treatment protocols.

## 5. Therapeutic Interventions and Translational Prospects

Despite growing recognition of the role of cellular senescence in the pathophysiology of IUGR, therapeutic interventions directly targeting senescent pathways during pregnancy remain underexplored. However, the increasing understanding of senescence mechanisms provides a unique opportunity to develop and repurpose therapeutic strategies that may mitigate placental dysfunction and improve fetal outcomes.

### 5.1. Senotherapeutics in Pregnancy

Senotherapeutics come in two classes: senolytics, which selectively eliminate senescent cells via suppression of anti-apoptotic pathways (e.g., BCL-2, BCL-W, and BCL-XL proteins) [133], and senomorphics, which suppress the harmful secretory phenotype (SASP) without inducing cell death [134]. Several senolytic compounds—such as dasatinib, quercetin, and navitoclax (ABT-263)—have demonstrated efficacy in aging and disease models, including models of idiopathic pulmonary fibrosis, osteoarthritis, and cardiovascular aging [135,136]. While these agents have not yet been evaluated in pregnancy due to safety concerns, their use in short, time-restricted windows, such as during the late second or early third trimester, may be a viable strategy worth investigating in preclinical models.

### 5.2. Rytvela as a Potent Senotherapeutic in IUGR and Preterm Birth

The SASP factors promote inflammation and immune cell recruitment in the placenta, exacerbating tissue dysfunction. Anti-inflammatory agents such as IL-1 receptor antagonists, p38 MAPK inhibitors, or NF-κB inhibitors may mitigate SASP-induced pathology [134]. These approaches could be particularly relevant for IUGR cases associated with maternal immune dysregulation, such as preeclampsia and preterm birth.

**Figure 3:**
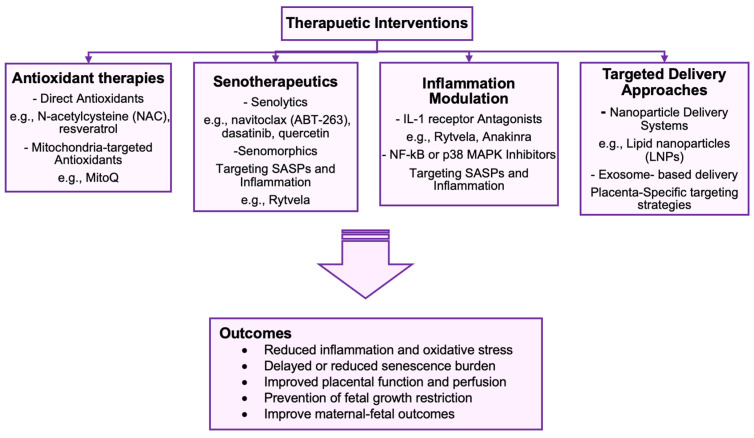
Overview of therapeutic interventions targeting cellular stress and senescence pathways implicated in IUGR. The figure summarizes four major strategies: antioxidant therapies, senotherapeutics (senolytics and senomorphics), inflammation modulation, and targeted delivery approaches. These therapies aim to improve placental function, reduce oxidative and inflammatory stress, restore telomere integrity, and ultimately improve fetal growth and maternal–fetal outcomes.

Rytvela is a small-molecule allosteric modulator of the interleukin-1 receptor (IL-1R) that was developed to selectively inhibit pro-inflammatory signaling without impairing essential immune functions [137]. Unlike classical IL-1 blockers such as anakinra, Rytvela preserves innate immune responses by sparing MyD88-dependent pathways essential for host defense, notably the NF-κB pathway, while selectively suppressing the inflammatory signaling cascade of p38 MAPK and ROCK [137,138]. This targeted immunomodulation makes Rytvela particularly appealing for pregnancy-related disorders where immune balance is critical, such as in IUGR, preeclampsia, and preterm birth (Figure 3). In preclinical studies, Rytvela displayed a potent anti-inflammatory effect in models of preterm birth, reducing systemic cytokine levels, prolonging gestation, markedly diminishing fetal/newborn mortality, and protecting against neurodevelopmental damage [139,140]. In pregnant sheep, Rytvela significantly reduced chorioamnionitis [141]. Given the established role of inflammation and the SASP in senescence-driven placental dysfunction, Rytvela may offer a novel therapeutic strategy as a senomorphic drug to attenuate inflammation-mediated senescence in trophoblasts. Rytvela’s favorable safety profile and selectivity make it a promising candidate for translational application in obstetrics. Ongoing development of placenta-specific delivery systems and time-controlled release platforms could further enhance its applicability during gestation.

### 5.3. Antioxidant and Mitochondrial Therapies

Given that oxidative stress is a key driver of senescence in trophoblasts, antioxidant-based therapies have shown promise in alleviating placental dysfunction. Agents such as N-acetylcysteine (NAC), resveratrol, and mitochondria-targeted antioxidants like MitoQ have been shown to reduce oxidative damage and delay senescence in vitro and in animal models [142,143,144]. However, clinical translation has been limited, and further studies are needed to evaluate their efficacy and safety in high-risk pregnancies. These potential therapeutics can be further enhanced by targeted distribution to the placenta, using, for instance, lipid nanoparticles (LNPs) or exosome-based delivery, which offer a promising avenue to limit systemic exposure and enhance fetal safety [145,146] (Figure 3).

## 6. Conclusions

IUGR presents formidable challenges in perinatal medicine, significantly impacting both short- and long-term health outcomes. The interconnection between fetal development and cellular senescence, particularly within the context of oxidative stress, provides crucial insights into the etiology and potential interventions for these conditions. Telomeres, essential for chromosomal stability, play a critical role in linking prenatal conditions to future health. Evidence suggests that telomere shortening and dysfunctional telomere maintenance mechanisms, such as reduced telomerase activity, are implicated in IUGR. Notably, oxidative stress not only exacerbates these processes but can also act as a direct driver of telomeric damage and telomerase downregulation, while transiently promoting alternative lengthening of telomeres, further amplifying cellular dysfunction. These interrelated mechanisms contribute to the pathology of IUGR by disrupting major growth factor signaling pathways, particularly the insulin and IGF pathways.

The role of cellular senescence in both physiological and pathological contexts during pregnancy highlights its dual nature. While senescence contributes to normal embryonic development, its aberrant induction due to stressors like oxidative damage can lead to adverse pregnancy outcomes, including IUGR and PE. Understanding the molecular underpinnings of these processes is crucial for developing therapeutic strategies to mitigate the risks associated with IUGR. Future research should focus on elucidating the precise molecular and cellular mechanisms driving telomere dynamics, oxidative stress responses, and senescence pathways in the context of IUGR. Additionally, developing diagnostic tools, such as non-invasive techniques for in vivo assessments of telomerase activity or senescence markers, using circulating cell-free nucleic acids, exosomal cargo, or advanced imaging approaches and exploring potential interventions to modulate these processes holds promise for improving maternal and fetal health outcomes, ultimately reducing the burden of growth restriction and the ensuing complications.

## Data Availability

No new data were created or analyzed in this study.
